# ELISA detection of SARS-CoV-2 antibodies in saliva

**DOI:** 10.1038/s41598-020-77555-4

**Published:** 2020-11-30

**Authors:** Melanie A. MacMullan, Albina Ibrayeva, Kylie Trettner, Laura Deming, Sudipta Das, Frances Tran, Jose Ricardo Moreno, Joseph G. Casian, Prithivi Chellamuthu, Jeffrey Kraft, Kenneth Kozak, Fred E. Turner, Vladimir I. Slepnev, Lydia M. Le Page

**Affiliations:** 1 Curative Inc, 430 S Cataract Ave, San Dimas, CA 91773 USA; 2 Mork Family Department of Chemical Engineering and Materials Science, Viterbi School of Engineering, University of Southern California, Los Angeles, USA; 3 Eli and Edythe Broad Center for Regenerative Medicine & Stem Cell Research at USC, Department of Stem Cell Biology and Regenerative Medicine, W.M. Keck School of Medicine, Los Angeles, USA; 4 Davis School of Gerontology, University of Southern California, Los Angeles, CA 90089 USA; 5 Bridge Institute, Loker Hydrocarbon Research Institute and Department of Chemistry, University of Southern California, Los Angeles, USA

**Keywords:** Viral infection, Diagnostic markers, ELISA

## Abstract

To facilitate containment of the COVID-19 pandemic currently active in the United States and across the world, options for easy, non-invasive antibody testing are required. Here we have adapted a commercially available, serum-based enzyme-linked immunosorbent assay (ELISA) for use with saliva samples, achieving 84.2% sensitivity and 100% specificity in a set of 149 clinical samples. This strategy will enable widespread, affordable testing for patients who experienced this disease, whilst minimizing exposure risk for healthcare workers.

## Introduction

Following the first confirmed case of the 2019 coronavirus (SARS-CoV-2) in Wuhan, China on December 2, 2019^1^, cases spread both within China and worldwide, with the World Health Organization declaring a pandemic on March 11, 2020^2^. 89,625 cases have been reported in China as of August 14 2020^3^. Worldwide, as of that same date, more than 20 million cases and over 750,000 confirmed deaths have been reported. Approximately a quarter of these cases and a fifth of these deaths have occurred in the United States, following the first known case in the US reported on 19 January 2020 in Washington State^4^. The continuing, devastating presence of this virus in the US makes it essential that the best tools are developed to understand this disease in a large and diverse population.

Taking into account our current understanding of previous coronaviruses, it is expected that the majority of patients with SARS-CoV-2 will develop antibodies against the virus. The emerging literature supports this, although our understanding of the rates of seroconversion and timelines of immunoglobulins M, G, and A is still developing^5^. Antibody detection is typically performed using an enzyme-linked immunosorbent assay (ELISA) on patient serum samples. Collection of these samples requires the use of trained phlebotomists which limits the ability to test a large patient population. Further, in collecting these samples, healthcare professionals are then exposed to potentially infectious environments. Alternative samples for testing would be valuable since they might enable more widespread testing that is safer for healthcare workers.

In testing for the SARS-CoV-2 virus in the US, the Centers for Disease Control and Protection have expanded their recommended sample type options to include several upper respiratory tract specimen types^6^. This variety of specimen options provides greater opportunity for widespread testing, and from these options, the easiest methods of sampling would enable widespread and frequent testing. The same principle applies to antibody testing.

Although serum is the typical sample type for detecting developed antibodies against many infectious diseases, dried blood spots^7,8^ and saliva samples^9–11^ have also been used successfully. Saliva samples are particularly attractive given the ease and non-invasiveness of sampling, allowing for potential self-collection and substantial scaling of testing. IgG antibody profiles have been shown to be similar in blood and saliva, with antibody titer for Hepatitis B correlating well between plasma and saliva^11^. Further, for IgG antibodies against human immunodeficiency virus type 1 (HIV-1), saliva samples correctly identified 102 of the 103 seropositive individuals tested^10^.

This work suggests that it is a valid strategy to explore saliva as an alternative to serum for detecting antibodies against SARS-CoV-2. Initial discussion^12,13^ and testing in small sample sets also supports further investigation of this avenue of research^14^.

In this manuscript we hypothesized that saliva is an acceptable specimen for the detection of SARS-CoV-2 antibodies by standard ELISA. To test this, we first assessed the sensitivity and specificity of two commercially available assays for serum (following manufacturers’ instructions). We then investigated multiple strategies for adapting the protocol of our chosen assay with the goal of detecting antibodies in saliva. For this investigation, paired serum and saliva samples were collected from individuals who were determined to be both positive for SARS-CoV-2 by RT-qPCR, and antibody positive by serum testing.

We conclude that, when following our optimized protocol, it is possible to detect antibodies against SARS-CoV-2 in saliva samples with a sensitivity of 84.2% and a specificity of 100% in a general symptomatic population. If this population is limited to those over the age of 40, sensitivity of 91.5% and specificity of 100% is achievable.

## Results

**Figure 1:**
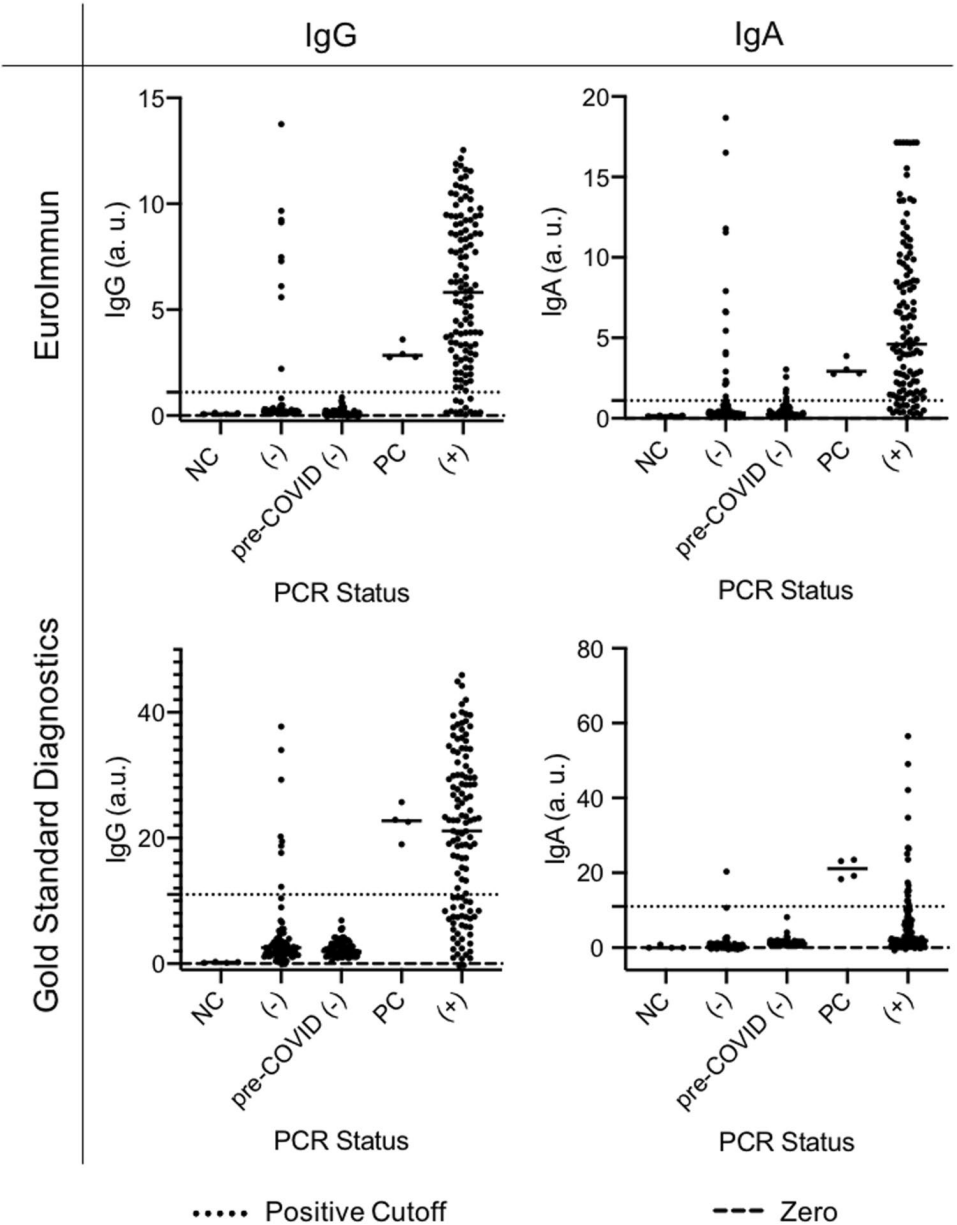
Sensitivity and specificity of four serum-based ELISAs: Detection of IgG or IgA antibodies against SARS-CoV-2 in serum samples collected prior to the spread of COVID-19 (pre-Nov. 2019), and people who previously tested negative (−) or positive (+) for COVID-19 using Curative's oral fluid PCR test. The positive cutoff values represented by the dotted lines were provided by the manufacturer. NC = negative control; PC = positive control.

Commercially available SARS-CoV-2 serum-based antibody detection ELISA kits from Gold Standard Diagnostics (GSD) and EuroImmun (EI), which detect the nucleocapsid (N) and spike (S) SARS-CoV-2 structural proteins respectively, were evaluated for their efficacy in detecting IgA and IgG antibodies against SARS-CoV-2 in clinical serum samples (Fig. 1). Due to uncertainty about which antibodies are most persistent over time^15^, kits for detecting both IgA and IgG antibodies were evaluated. Kits for detecting IgM antibodies were also considered, but due to a lack of consensus surrounding the persistence of IgM in blood^16^ we chose to pursue IgG and IgA antibodies only. 76 serum samples collected prior to November 2019 (pre-SARS-CoV-2) were used as a negative control panel to evaluate specificity for both IgG and IgA kits from each manufacturer. Each sample was run in singlicate, and whilst samples may have been run across multiple days, each kit contained a calibrator which was used to normalize antibody signal for cross-plate comparison. We determined that both GSD IgG and IgA kits and the EI IgG kit were 100% specific, while the EI IgA kit was 92% specific. We then also tested these kits for sensitivity against clinical serum samples collected between April and July 2020.

Because of limited and confounding existing data on asymptomatic patients and the increased sensitivity 21 days post-symptom onset reported by the ELISA manufacturers, we chose to only investigate samples from symptomatic participants collected more than 21 days post-symptom onset. We tested serum collected from 123 symptomatic, PCR-positive patients and from 83 PCR-negative patients on both IgG and IgA ELISA kits from each manufacturer (Fig. 1). Based on the virus-positive patient samples run in singlicate, EI IgG and IgA kits were 90 and 86 percent sensitive respectively and GSD IgG and IgA kits were 69 and 15 percent sensitive respectively based on cutoff values for serum supplied by the manufacturers. Nine participants who had tested PCR-negative for RNA derived from SARS-CoV-2 were positive for IgG antibodies against the virus based on both EI and GSD antibody detection kits, suggesting that these patients were likely infected by COVID-19 prior to their RT-qPCR test (Supp. Fig. 1).

**Figure 2:**
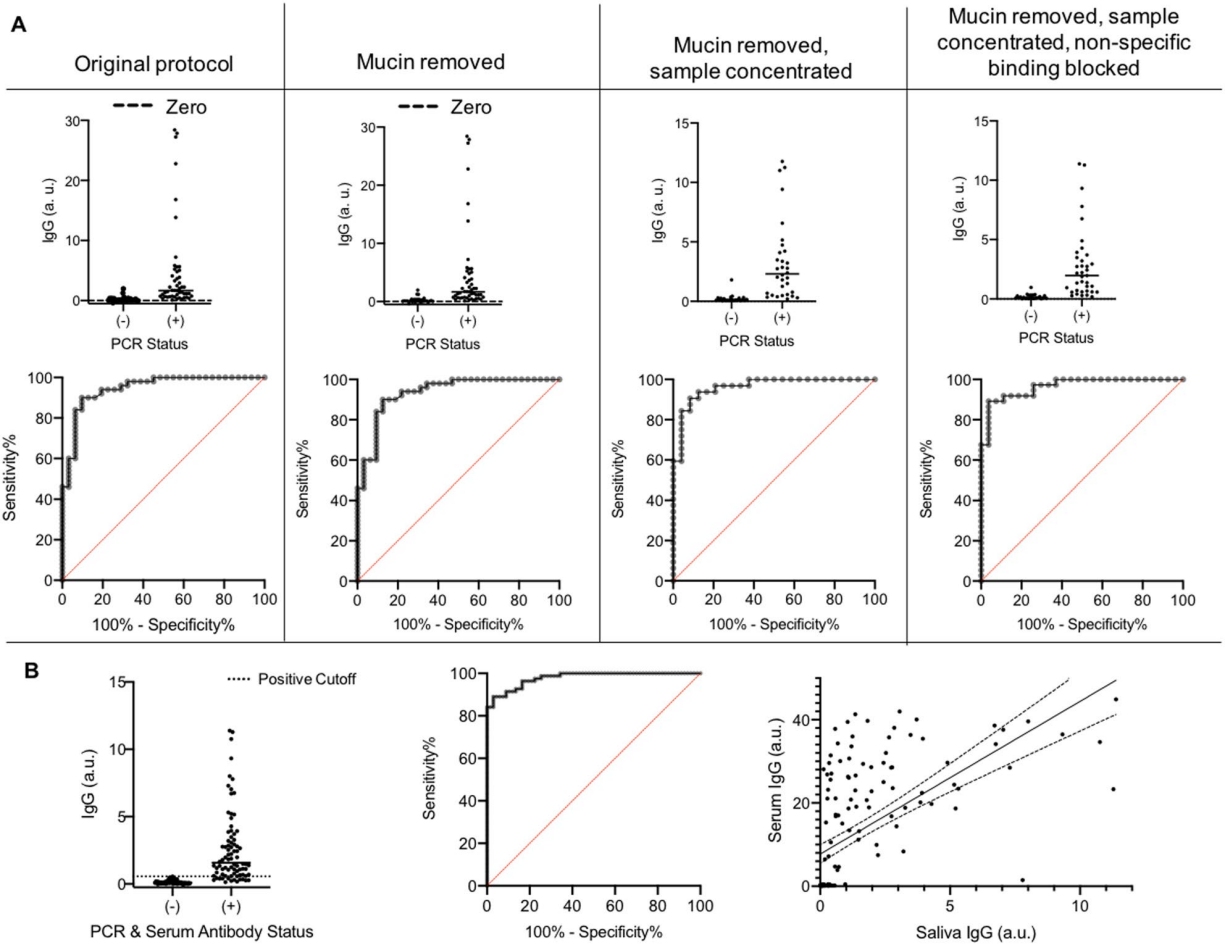
Investigation of protocol changes to enable use of saliva samples: (**A**) Sample conditions tested for optimized detection of anti-SARS-CoV-2 antibodies in mouthwash saliva samples, in comparison to the original protocol (AUC = 0.946): mucin removed (AUC = 0.935), mucin removed and sample concentrated (AUC = 0.962), and mucin removed. Sample concentrated, and non-specific binding blocked (AUC = 0.965). (**B**) IgG was detected using the optimized protocol in a large sample set where positive and negative status was determined by both PCR and serum antibody status, providing 100% specificity with 84.2% sensitivity (AUC = 0.979). A Pearson correlation showed a weak but significant correlation between serum and saliva IgG (R = 0.596, p < 0.0001). Line of best fit is displayed with 95% confidence intervals.

Our objective was to evaluate the ability of these tests to detect antibodies in saliva in addition to detecting antibodies in serum samples. We selected the EI IgG kit for our saliva sample optimization experiments based on its superior performance in our clinical serum sample testing. All clinical study participants provided saliva and serum samples for antibody testing. Saliva samples were collected using two devices as described in the Methods section. We selected saliva samples from COVID-19 positive participants (as determined by RT-qPCR) that were also positive for antibodies in serum, and samples from COVID-19 negative participants (as determined by RT-qPCR) that were also negative for antibodies in serum. Interested in comparing two different methods of collecting saliva, we compared the OraSure Technologies Oral Fluid Specimen Collection Device to a mouthwash prepared from an inhouse formulation (detailed in Methods section) using a subset of saliva samples (Supp. Fig. 2). Each sample was run in singlicate and we determined that the mouthwash yielded 100% sensitive and specific results for antibody detection while the OraSure Collection Device yielded only 87% sensitivity and 100% specificity. Based on this assessment, we chose to go forward with the mouthwash. Increasing our sample size to 50 positive and 33 negative samples reduced the specificity to 93.6% and the sensitivity to 84.0% with an area under the receiving operator characteristic (ROC) curve (AUC) of 0.946 (Fig. 2A).

Based on this data, we further optimized the sample preparation and ELISA processes. Samples run across multiple plates were calibrated using a manufacturer-provided calibrator solution. We first tested centrifugation of the samples to remove a large pellet of mucin, a common practice among studies of whole saliva samples^11,17^. This gave a sensitivity of 84.0% and specificity of 90.6% (AUC = 0.935). We then added a concentration step (Amicon Ultra Centrifugal Filters) which improved sensitivity to 84.4% and specificity to 95.8% (AUC = 0.962). Finally, we blocked the ELISA plate with a blocking buffer containing TBS-Tween and 5% BSA for 30 min before adding sample, yielding an optimal sensitivity of 89.2% and specificity of 96.3% (AUC = 0.965) with a positivity cutoff of 0.40; this final protocol yielded the best sensitivity and specificity for this set of mouthwash samples (Fig. 2A).

To determine whether the test remained sensitive and specific in an expanded population, we applied this combined protocol (i.e. centrifugation, concentration, and blocking of the ELISA plate) to a larger mouthwash sample group of 82 positive and 67 negative samples. In this population, with the positivity threshold maintained at 0.40, we saw 86% sensitivity and 97% specificity. As a final optimization step, and to be used in all future work, we would propose the positivity threshold be set at 0.56, giving 84.2% sensitivity and 100% specificity (AUC = 0.979), so no false positives are reported (Fig. 2B). Antibody detection in paired saliva and serum samples showed a weak but statistically significant correlation (R = 0.596, p < 0.0001). (Fig. 2B).

**Figure 3:**
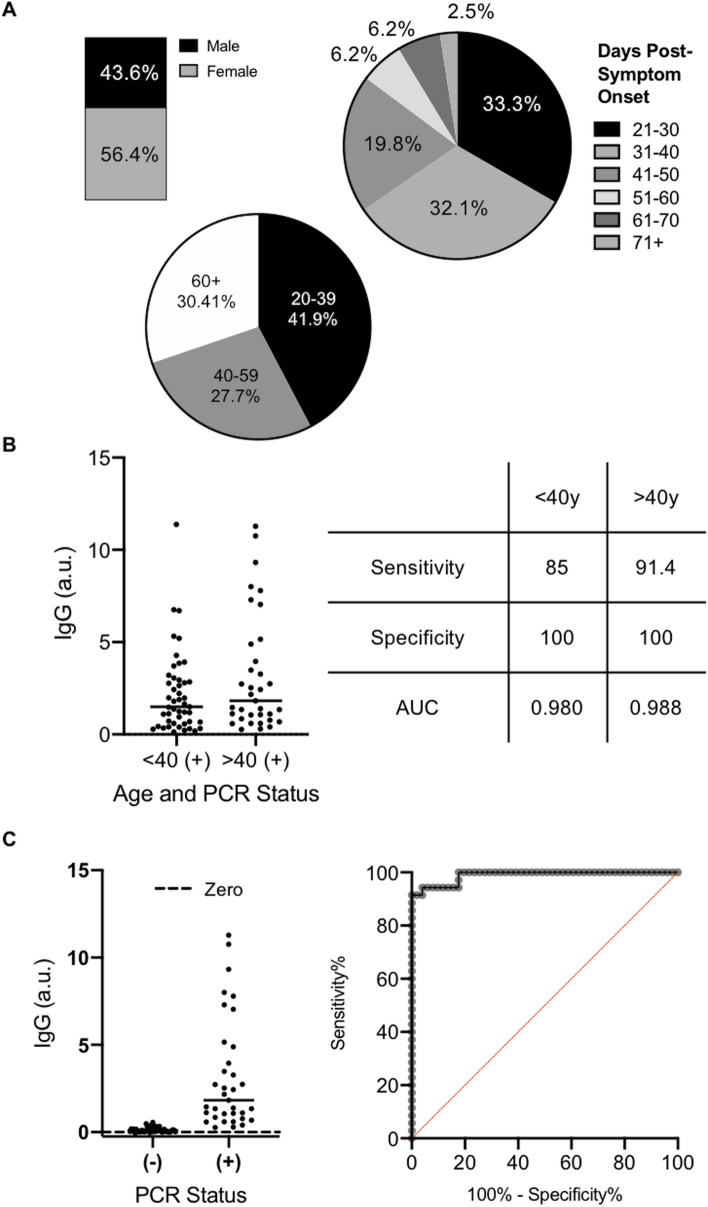
Investigating the optimal population for a saliva-based ELISA: (**A**) Visualization of the gender (n = 149), age (n = 149), and days post symptom onset (n = 85) of our cohort of clinical samples. (**B**) Stratifying by age impacted the sensitivity and specificity of our optimized saliva protocol. (**C**) When limited to sampling people over 40 years of age (n = 86), 91.5% sensitivity and 100% specificity (AUC = 0.988) can be achieved.

Given our cohort of clinical samples (demographics in Fig. 3A), we wanted to determine if factors linked to COVID-19 susceptibility impacted our sensitivity and specificity. By stratifying the data based on patient demographics of age, sex, and days elapsed since onset of symptoms for sample collection (Supp. Table 1), we found that testing samples only from those over 40 years of age further improved our saliva-based antibody detection test (Fig. 3B). This sample set gave a sensitivity of 91.5% and specificity of 100% (AUC = 0.989, Fig. 3C). This optimized test could be used on populations of patients of 40 years of age or more, where we might suggest a borderline range between IgG ratios of 0.40 and 0.55, and consider positive tests having an IgG ratio greater than 0.56.

## Discussion and conclusion

We have shown that testing for IgG against SARS-CoV-2 is possible in saliva samples, providing an easy, noninvasive option for detection of prior infection. Further optimization and increasing the cohort size may improve this method to the levels recommended by the FDA guidance (better than 90% sensitivity and 100% specificity).

It is interesting to note our increased AUC when limiting our testing population to individuals over 40 years of age. It has been established that the risk for severe disease increases with age^18^. Seow et al*.*^19^ have described the size of neutralizing antibody response as being dependent on disease severity, and Long et al*.*^15^ have described significantly lower virus-specific IgG levels in asymptomatic individuals compared with symptomatic. This may explain our results, although it is important to note that this link is not confirmed, with Phipps et al*.*^20^ seeing no association between IgG level and severity of disease in their patient population. Further, these studies are all investigating serum IgG; saliva IgG may present differently and has yet to be fully investigated. Finally, further work is required to describe results in asymptomatic COVID-19 positive people, estimated to be as high as 40% of cases^21^.

There is limited literature available investigating saliva samples for SARS-CoV-2 antibody testing. However, work by Randad et al*.*^14^ developing a multiplex immunoassay supports the outcomes of our study, demonstrating a significant correlation between SARS-CoV-2 IgG serum and saliva levels in samples from 22 PCR positive and 6 PCR negative individuals. Their work also tests the sensitivity and specificity of testing for IgG in saliva, although reports that sensitivity does not exceed 57% in their most comparable S1-antigen assay. This may be due to, amongst other factors, the origin of the antigen used in the assay or the population of individuals tested—samples in the manuscript by Randad et al*.*^14^ were greater than 10 days after symptom onset whereas our population was later in the disease course (> 21 days after symptom onset).

We also investigated IgA as a potential biomarker of SARS-CoV-2 infection. Due to worse performance in the serum ELISA in both EI and GSD assays, and given we had a limited volume of saliva samples with which to optimize, we did not pursue this further. Indeed, although Randad et al*.*^14^ showed good specificity for IgA, sensitivity did not exceed 61%. However, more recent work by Varadhachary et al.^22^ reports a rapid immunoassay designed for point-of-care use that showed 92% positive agreement and 97% negative agreement in 36 and 32 samples respectively.

Moving forward, implementation of our optimized protocol in a clinical lab is possible in order to scale testing. Given the ease of sample collection and low cost of the mouthwash, large populations could be tested easily. The ELISA is an inherently scalable, high throughput assay that has been fully automated for a range of serum-based assays by multiple companies. Our additional protocol steps are straightforward and so should not limit scaling; the additional step of plate blocking could be managed offsite rather than in the clinical lab space. Thus, the two centrifugation steps (both requiring the same equipment) are the only additional processes to this well-established assay.

Development of this specific, sensitive antibody test from non-invasive samples which require no exposure of healthcare workers to potentially infectious environments may be very valuable. Amongst the millions of cases of COVID-19, there are currently limited reports of documented reinfection in the US within 3 months of initial infection^23^; immunity may be protective on this timescale. Antibody testing could provide significant information for countries interested in establishing the reach of the disease in order to help make policy decisions about reopening towns, cities, and states.

## Methods

### Human serum and saliva sample collection

#### Pre-COVID-19 serum samples

These samples were purchased from Cureline (Brisbane, CA), and were collected before September 2019 from healthy adults in the USA.

#### Post-COVID-19 serum and saliva samples

Clinical samples were collected under UCLA Institutional Review Board approved study protocol IRB#20-000703. The UCLA IRB determined the protocol was minimal risk and verbal informed consent was sufficient for the research under 45 CFR 46.117(c)(2). The study team complied with all UCLA policies and procedures, as well as with all applicable Federal, State, and local laws regarding the protection of human subjects in research as stated in the approved IRB.

For this study, we worked with four specimens obtained: oral fluid for viral RT-qPCR testing, blood via venipuncture, and two saliva specimens.

#### Oral fluid swab for viral RNA

This was obtained according to the reported guidelines for Curative’s oral fluid COVID-19 test. Briefly, participants coughed hard three times while shielding their cough via mask and/or coughing into the crook of their elbow. They then swabbed the inside of their cheeks, along the top and bottom gums, under the tongue, and finally on the tongue, to gather a sufficient amount of saliva. Swabs were placed in a tube containing RNA Shield and transported at room temperature before laboratory processing as described^24^. RT-qPCR was performed to determine positive and negative samples. Positive for viral RNA was determined as below 35 cycle threshold (CT).

#### Blood sampling

Participants underwent a standard venipuncture procedure. Briefly, licensed phlebotomists collected a maximum of 15 ml whole blood into 3 red-top SST tubes (Becton–Dickinson, cat. number 367988). Once collected, the sample was left at ambient temperature for 30–60 min to coagulate, then was centrifuged at 2200–2500 rpm for 15 min at room temperature. Samples were then placed on ice until delivered to the laboratory site where the serum was aliquoted to appropriate volumes for storage at − 80 ºC until use.

#### Orasure saliva sample collection

Orasure oral specimen collection devices (catalog number 3001-2870, Orasure, USA) were used as instructed^25^. The pad was brushed briefly on the lower gums and then held between the gum and the cheek for 2–5 min. The pad was then placed into the storage tube, with the provided storage solution. Samples were kept on ice until they reached the lab. The samples were processed as recommended by the manufacturer^26^ before being aliquoted and stored at − 80 °C until use.

#### Mouthwash saliva samples

Mouthwash was made in-house (all chemicals from Sigma-Aldrich, USA) by adding 3% Sodium Chloride (15 g), 0.2% Citric Acid (1 g), 0.075% Sodium Benzoate (0.375 g), 0.075% Potassium Sorbate (0.375 g) to 500 mL of ddH_2_O. The solution was then autoclaved (121 ºC for 20 min) before pH adjustment to pH = 6.5 with 0.1 M of NaOH. 4 ml aliquots were then used to vigorously rinse the mouth for 1–2 min before being collected. Samples were kept on ice until they reached the laboratory, where they were aliquoted (avoiding any large particulates in the liquid) and stored at − 80 °C until use.

### Serum ELISA

EuroImmun SARS-CoV-2 IgA and IgG ELISAs^27^ for serum (cat. numbers EI 2606-9620 IgA, EI 2606-9620 IgG, EuroImmun, New Jersey, USA) targeting spike (S) protein were run according to the manufacturer provided protocol^28^ on the Thunderbolt (Gold Standard Diagnostics, Davis, CA) automated instrument. Briefly, serum was diluted 1:101 in each well with the provided sample buffer and then incubated at 37 ºC for 1 h. Sample wells were washed three times with a provided wash buffer (10× dilution with ddH_2_O, 0.35 ml per well), before the provided conjugate solution was added (0.1 ml per well) and incubated at 37 ºC for 30 min. After a second wash step, the provided substrate solution was added (0.1 ml per well) and incubated at ambient temperature for 30 min. The provided stop solution was then added (0.1 ml per well) and absorbance of sample wells measured immediately at 450 nm and 630 nm, with output reports generated with optical density (O.D.) at 630 nm subtracted from O.D. at 450 nm. Data were then analyzed as recommended by the manufacturer and results reported as a ratio (Eq. 1).

**Equation 1.** Determination of sample absorbance ratio based on sample O.D. divided by the averaged O.D. of the calibrators.

1 $$Ratio = \frac{O.D. \; of\;  the \; control \; or \; clinical \; sample}{O.D. \; of\; the\; calibrator}$$

Gold Standard SARS-CoV-2 IgG and IgA ELISAs for serum (cat. numbers GSD01-1029 IgA, GSD01-1028 IgG, Gold Standard Diagnostics, Davis, USA) targeting nucleocapsid (N) protein were run according to the manufacturer provided protocol on the Thunderbolt (Gold Standard Diagnostics, Davis, CA) automated instrument. Briefly, serum was diluted 1:101 in each well with the provided sample buffer and then incubated at room temperature for 30 min. Sample wells were washed three times with a provided wash buffer (20X dilution with ddH_2_O, 0.3 ml per well), before the provided conjugate solution was added (0.1 ml per well) and incubated at ambient temperature for 30 min. After a second wash step, the provided substrate solution was added (0.1 ml per well) and incubated at ambient temperature for 30 min. The provided stop solution was then added (0.05 ml per well) and absorbance of sample wells measured immediately at 450 nm and 630 nm, with output reports generated with optical density (O.D.) 630 nm subtracted from O.D. at 450 nm. Data were then analyzed as recommended based on a correction factor (specific to each kit) and mathematical formula provided by the manufacturer (Eqs. 2, 3).

**Equation 2.** Determination of sample positivity cutoff value as an average of the calibrator values multiplied by a lot-specific correction factor.

2 $$Cutoff = Correction\; Factor * \left(\frac{{O.D.}_{cal1}+{O.D.}_{cal2}}{2}\right)$$

Determination of the antibody units as defined by the manufacturer by dividing sample O.D. by the positivity cutoff value and multiplying by 10.

3 $$Units = \left(\frac{Sample \;O.D.}{Cutoff}\right)*10$$

### Saliva ELISA optimizations

#### Centrifugation of samples

1 ml of mouthwash samples were centrifuged at 15,000 rpm for 10 min (4 °C). The supernatant was then immediately transferred to a clean tube.

#### Concentration of samples

300 μl of supernatant was placed in Amicon Ultra Centrifugal Filters (UFC5199BK, Millipore Sigma, USA) and centrifuged at 4 °C, 15,000 rpm for 8 min. The concentrated sample was then recovered via a second centrifugation step (4 °C, 15,000 rpm for 5 min); this procedure produced ~ 50 μl of concentrated sample. 50 μl of a blocking buffer prepared in house (5% BSA in TBS with 0.5% Tween 20) was then added before the total volume (~ 100 μl) was placed in each well of the ELISA plate, and the protocol run as described above.

#### Blocking of ELISA plate

Prior to concentrated sample loading, 25 μL of the in house blocking buffer (5% BSA in TBS with 0.5% Tween 20) was added to each well. Wells were incubated with a blocking buffer for 30 min at room temperature before adding a sample to begin the ELISA.

### Statistical analysis

ROC curves were generated in GraphPad Prism (GraphPad Prism Version 8.4.3, San Diego, USA), with a 95% confidence interval. Area under the curves was also calculated. Correlation between serum and saliva IgG values were calculated using a Pearson correlation computing R between the two datasets, with a 95% confidence interval.

## Supplementary information

Supplementary Figure S1.

Supplementary Figure S2.

Supplementary Table S1.

## Data Availability

The full dataset that supports the findings of this study is available from the authors upon reasonable request.
