# Supplementary figures and images for: ELISA detection of SARS-CoV-2 antibodies in saliva
Source: Sci Rep. 2020 Nov 30;10:20818. doi: 10.1038/s41598-020-77555-4 (PMC7705674; doi:10.1038/s41598-020-77555-4)

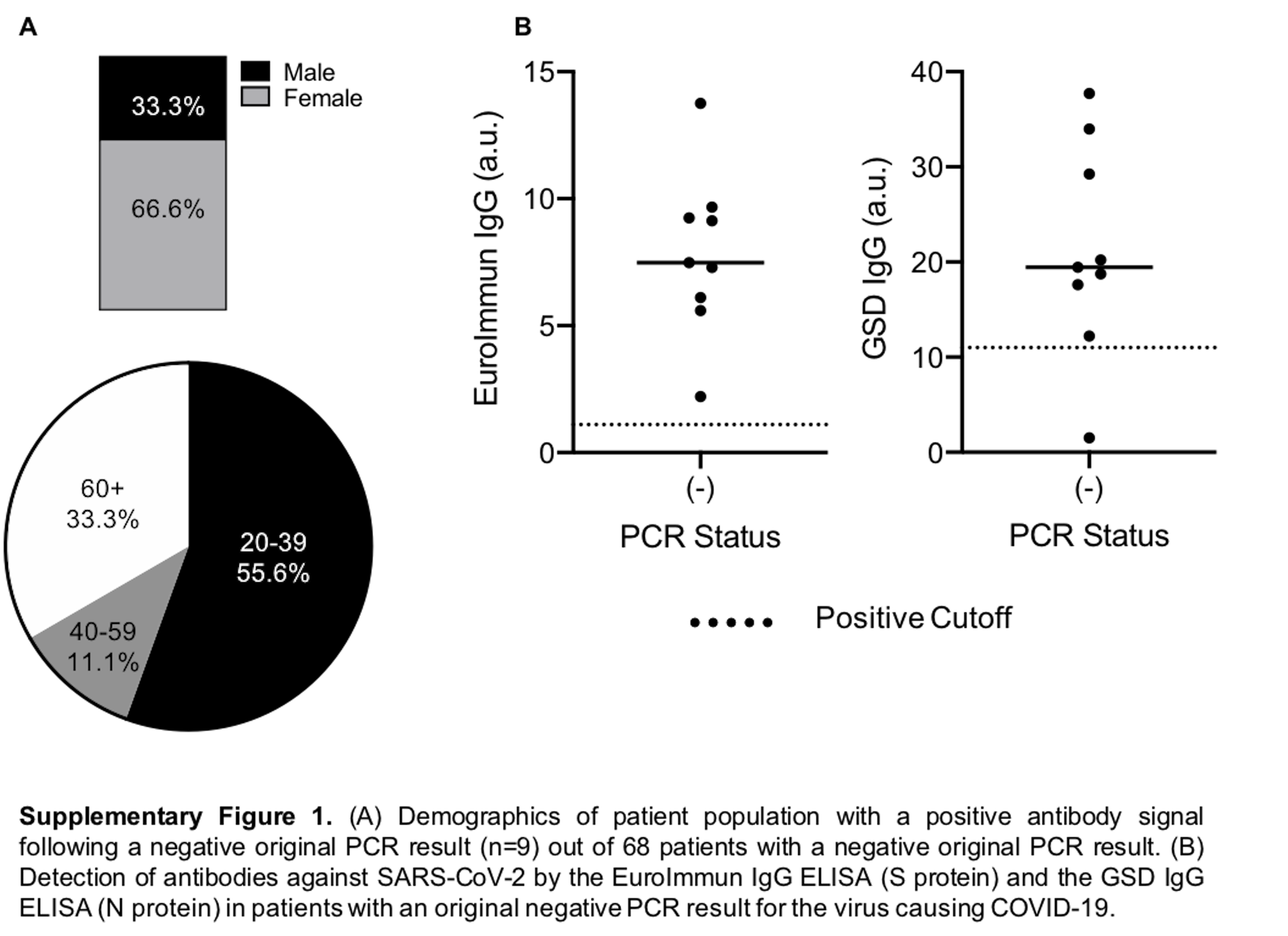

Supplement: Supplementary file 1 — Supplementary Figure S1. [file 41598_2020_77555_MOESM1_ESM.jpg]

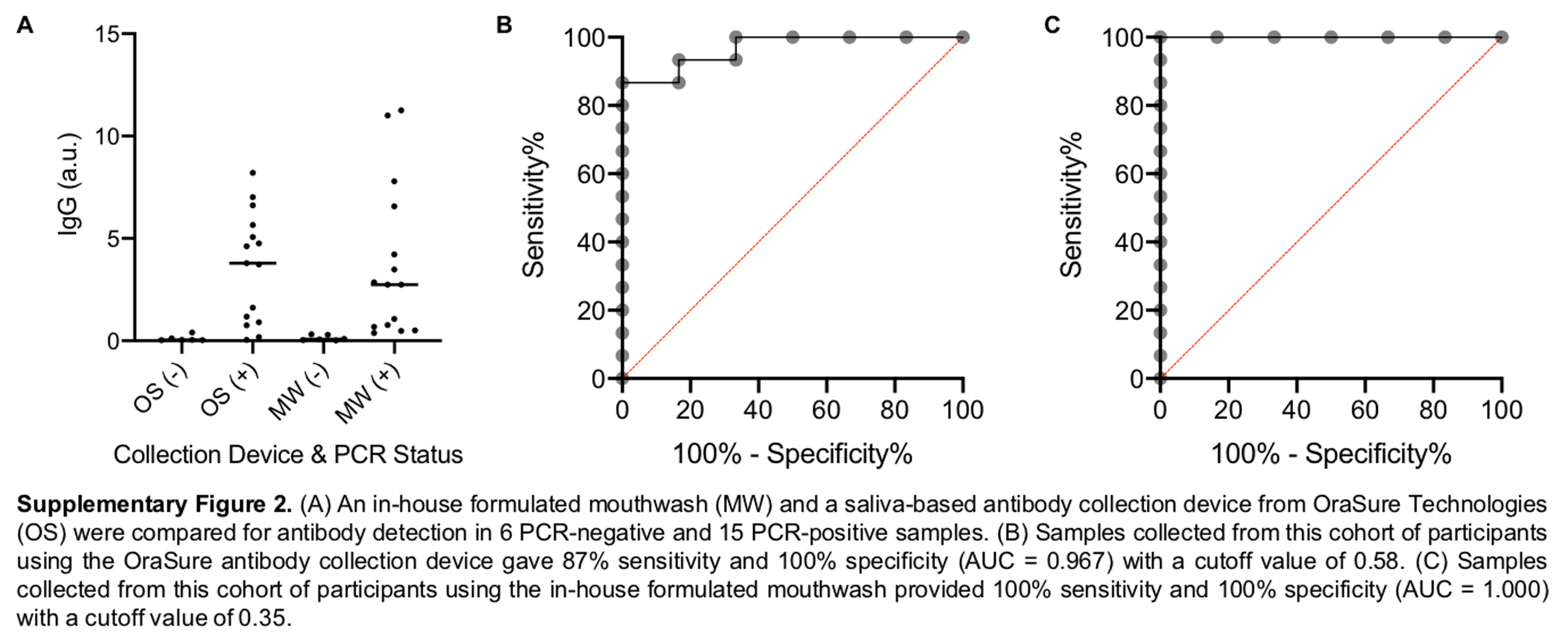

Supplement: Supplementary file 2 — Supplementary Figure S2. [file 41598_2020_77555_MOESM2_ESM.jpg]

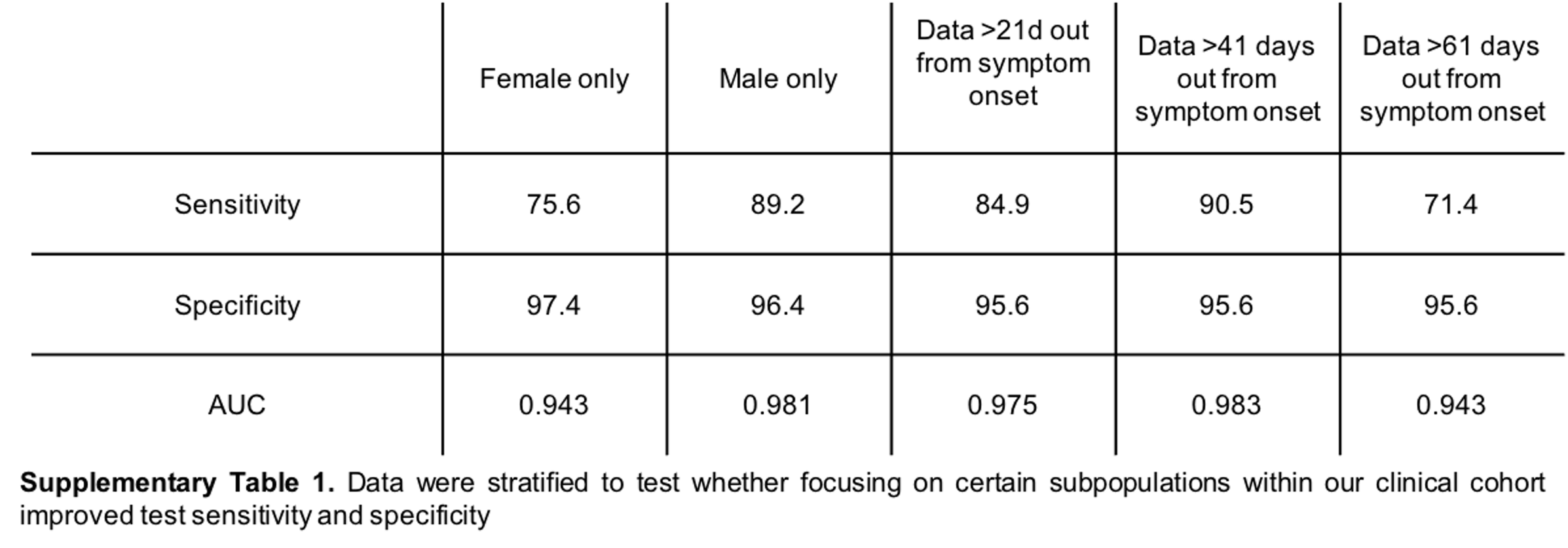

Supplement: Supplementary file 3 — Supplementary Table S1. [file 41598_2020_77555_MOESM3_ESM.jpg]
